# Clinical yarning education: development and pilot evaluation of an education program to improve clinical communication in Aboriginal health care - participant, and health manager perspectives

**DOI:** 10.1186/s12909-023-04843-8

**Published:** 2023-11-30

**Authors:** Ivan Lin, Wanda Flanagan, Charmaine Green, Anne Lowell, Juli Coffin, Dawn Bessarab

**Affiliations:** 1 Western Australian Centre for Rural Health, University of Western Australia, Fitzgerald St, Geraldton, WA 6531 Australia; 2 Research Centre for Health and Wellbeing, Charles Darwin University, Ellengowan Drive, Brinkin, Darwin, NT 0909 Australia; 3 Ngangk Yira Institute for Change, Murdoch University, Murdoch, WA 6150 Australia; 4 Centre for Aboriginal Medical and Dental Health, University of Western Australia, Stirling Highway, Crawley, WA 6009 Australia

**Keywords:** Indigenous, Cultural security, Safety, Competence, Training, Quality

## Abstract

**Background:**

Effective communication between health care clinicians and Aboriginal patients is critical to delivering high quality, accessible, culturally secure health care. Despite this, ineffective communication is a well-documented barrier, and few studies have reported interventions to improve communication. Clinical Yarning is a patient centred communication framework for Aboriginal health care. Building on this framework, this study reports the development and evaluation of a Clinical Yarning education program.

**Methods:**

A Clinical Yarning education program was developed, underpinned by the principles of cultural security and adult learning, informed by a behavioural skills approach. The program was delivered in five health/education settings in one rural Western Australian region. Mixed-methods evaluation included a retrospective pre/post questionnaire to ascertain changes in participants’ knowledge, confidence, competence and their perceptions about communication in Aboriginal health care, and the program. Qualitative semi-structured interviews were undertaken with health service managers who oversaw each health care setting and who had not participated in the education program, to explore perceptions about the program and implementation considerations.

**Results:**

Twenty-eight health care clinicians and six students completed training and the evaluation survey. There were significant improvements in self-rated communication skills, ability, confidence, knowledge, and perceived importance of communication training from pre to post-program. Participants strongly recommended the program to others, and most commonly valued the simulation/interactive learning activities. Health service managers acknowledged the limitations in most existing cultural training, and felt Clinical Yarning addressed a need; both the concept of Clinical Yarning and the education program provided were valued. Considerations identified for future implementation included: building multilevel partnerships within health services, offering alternate training options such as eLearning or train-the-trainer approaches, and integrating into existing development programs. Workforce transiency and availability were a barrier, particularly in remote areas.

**Conclusions:**

This study offers preliminary support for the Clinical Yarning education program and provides a foundation for further development of this training approach. A future priority is implementation research to investigate the impact of the Clinical Yarning education program on health care and patient outcomes.

**Supplementary Information:**

The online version contains supplementary material available at 10.1186/s12909-023-04843-8.

## Background

A critical step to reducing health disparities between Aboriginal and Torres Strait Islander (henceforth respectfully ‘Aboriginal’) and non-Aboriginal Australians is to improve the quality and cultural security of health care [1]. An increasingly recognised barrier to quality and culturally secure care (in which practices and policies that meet the needs of Aboriginal people are embedded within health care [2]) is ineffective communication between Aboriginal patients and health care practitioners (HCPs) [3–5]. Suboptimal communication between HCPs and Aboriginal patients has been extensively reported across a multitude of health areas including; cardiac conditions [4], diabetes [3], kidney disease [6], chronic diseases [7], cancer [8] and musculoskeletal pain [5, 9].

Effective communication is the foundation of high quality health care, resulting in more accurate, efficient, and supportive health care consultations, better outcomes for patients, and greater patient and practitioner satisfaction [10]. Communication is also integral to the accessibility of health care. In the 2012-13 Australian Aboriginal and Torres Strait Islander Health Survey, a significant number of Aboriginal Australians (7%) reported avoiding health care because of the way they were treated by health staff [11]. Ineffective communication is a primary barrier to successful management of health conditions [12] and a major reason why Aboriginal Australians choose to disengage with health care [9]. Barriers to effective communication include HCPs who are not culturally aware [8], differences between Aboriginal and Western/biomedical perspectives of health [4], the use of medical jargon [4, 5], a lack of communication [6], language barriers [3], prejudicial attitudes of practitioners [4] and inadequate/incomplete explanations about health and disease [7]. These communication issues foster mistrust of health care services and can have profound negative consequences on the health and wellbeing of Aboriginal people. Communication issues with HCPs are also reported by First Nations people in North America [13] and New Zealand Maori [14, 15].

To date the majority of research has reported on communication challenges. Little research has focussed on interventions to improve communication between HCPs and Aboriginal patients, despite being identified by HCPs as the most important unaddressed topic in cultural education [16]. This represents a significant gap. Successful interventions to improve communication in Aboriginal health are an opportunity to improve health care, and subsequently reduce the high burden of illness amongst Aboriginal communities.

**Fig. 1:**
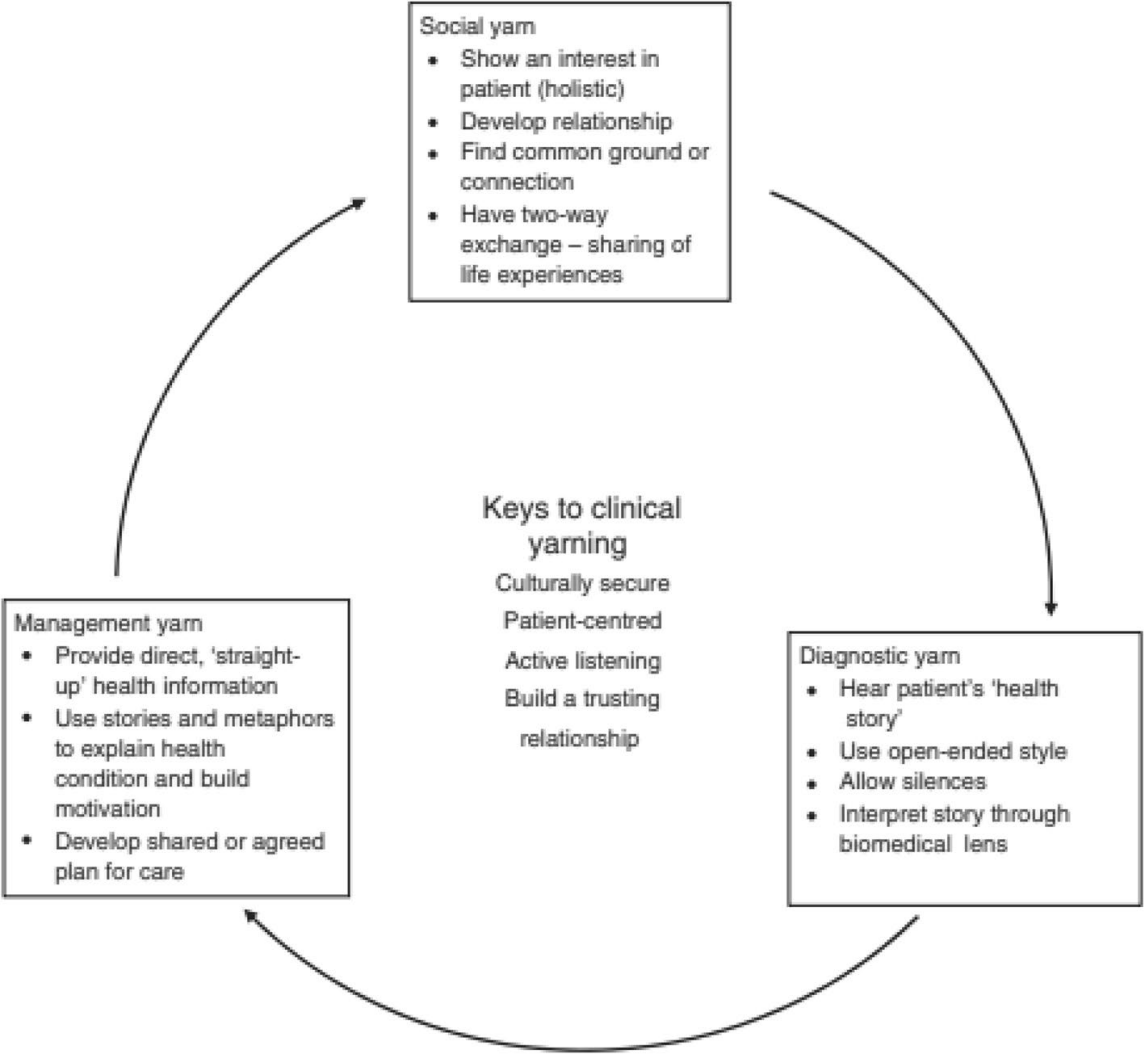
Key elements of the Clinical Yarning Framework, re-produced from [17]

Our team previously proposed a patient-centred communication framework for Aboriginal health care called ‘Clinical Yarning’ [17]. This framework originates from Bessarab and Ng’andu’s Research Yarning framework [18]. Clinical Yarning is a person-centred communication framework that uses a yarning approach to facilitate and engage with the patient’s story and health concerns in a friendly and culturally appropriate manner. Yarning is a form of communication utilised by Aboriginal Australians that is informal, two-way, and often involves exchange of information via storytelling [17]. Clinical Yarning provides practitioners with skills and tools to communicate more effectively by re-conceptualising clinical communication as a social, diagnostic and management yarn (Fig. 1). The process of Clinical Yarning focusses on establishing trust and connectedness with patients, and understanding a person’s health concerns by listening to their story. It enables the clinician to explain health information in culturally and contextually meaningful ways that engage patients, and their family, in treatment decision making. This article reports a first step to translate Clinical Yarning into Aboriginal health care practice; the development and preliminary evaluation of a ‘Clinical Yarning Education’ program for HCPs in rural and remote Western Australia.

## Methods

The project was undertaken in one rural/remote region of Western Australia including a regional location (Modified Monash (MM) Category 3 – large rural town, population approximately 30,000, 10% identify as Aboriginal), and remote (MM 7 – very remote, population approximately 700, 40% identify as Aboriginal). The project involved; (i) developing a Clinical Yarning education program, and (ii) delivering and evaluating the program from the perspective of health practitioners/students and health care managers.

Initially two project advisory groups were formed. The first was an ‘expert content group’. This group included Aboriginal and non-Aboriginal members with expertise in Aboriginal health, Aboriginal health research, health care, education, and patient-centred communication. The second advisory group consisted of Aboriginal representatives from different Western Australian regions who worked for one of the project’s main funding partners (WA Country Health Services).

### Developing a clinical yarning education program

Development of the Clinical Yarning education program involved (i) identifying key skills underpinning the Clinical Yarning approach, (ii) development of learning objectives, learning modules, a learning plan and learning activities. Learning activities were supported with audio-visual and audio case studies.

Key skills used in Clinical Yarning were identified by the expert content group through discussion and consensus. Following this, these skills were mapped against an established, skills-based patient-centred communication framework, the Calgary-Cambridge guide (CCG) [10]. This enabled our team to identify skills unique to Clinical Yarning, and skills in which there may be some overlap with existing patient-centred communication skills and therefore may be familiar to clinicians. Although there was overlap, our team observed that in some cases, a Clinical Yarning approach involved a different emphasis. For example, a strong focus of Clinical Yarning is working with a person’s story and understanding how their health issue relates to their story and their social-cultural lifeworld as an Aboriginal person. In contrast the overlapping skill in the CCG is to “encourage the patient to tell the story of the problem(s)”, placing a stronger emphasis on the health issue rather than the person. These nuances were noted. These skills are outlined in Table 1.

**Table 1:** Skills utilised in a clinical yarning approach. Skills with some overlap with CCG skills are identified (O-CCG)

**Social Yarn**
Key skills: • respectfully introducing oneself to the patient (O-CCG) • attending to the patient’s comfort (O-CCG) • welcoming body language (O-CCG) • finding common ground • sharing information about oneself • demonstrating awareness/knowledge of Aboriginal culture • where appropriate, working effectively with Aboriginal cultural mediators such as an interpreter • where appropriate, using humour • recognising verbal and non-verbal patient cues for the social yarn
**Diagnostic Yarn**
Key skills: • active/deep listening to the patient’s story (O-CCG) • facilitating the patient’s story e.g. through sensitive/responsive open-ended questioning (O-CCG) • use of silence (O-CCG) • recognising verbal and non-verbal patient cues (O-CCG) • validating the patient’s perspective (O-CCG) • summarising, prompting, and clarifying (O-CCG) • demonstrating empathy (O-CCG)
**Collaborative Management Yarn**
Key skills: • understanding what the patient knows about their health issue (O-CCG) • explaining health information without medical jargon (O-CCG) • using explanatory aids such as stories, metaphors, visual aids and online resources (O-CCG) • checking-in with what the patient understands and will take-away with them (O-CCG)

Following this, learning objectives, learning modules, and education methods for face-to-face delivery of a workshop or future eLearning program were developed. This was based on the skills identified in the previous step, underpinned by adult learning principles and a behavioural skills approach to clinical communication education. Adult learning principles assume that learning is most effective when learners identify a need to learn, when they engage in interaction with other learners, and when they have repeated opportunities to apply theory and information to relevant practical situations [19]. Like adult learning, behavioural skills approaches to learning emphasise skills acquisition via a process of experiential learning, involving repeated practice, reflection and rehearsal. These approaches are consistent with evidence that clinical communication skills are most effectively taught using active learning strategies such as role-play, reflective feedback, and small group discussion [20, 21]. Learning objectives, learning modules, and education methods were initially drafted by two team members (IL, WF) and refined through discussion with the expert content group.

The learning objectives, learning modules, and methods are outlined in Table 2. Modules 1–4, and module 5, were delivered in two, 2-hour sessions. A professional filmmaker was engaged to record short audio-visual and audio case studies to support learning activities. These included Aboriginal community members describing health care communication experiences, patient-practitioner health care consultations, and simulated examples of Clinical Yarning in health care consultations. Module 5 included simulated learning with Aboriginal simulation patients. These were Aboriginal community members (one male, one female) who received training in the Clinical Yarning model, the role of a simulation patient, and orientation to simulation scenarios. Module 5 applied the Agenda Led Outcomes Based Analysis (ALOBA) method [20], an approach and set of strategies for analysing and giving feedback in clinical communication experiential learning. ALOBA is learner-focused and aims to create a safe learning environment to improve communication skills.

**Table 2:** Clinical yarning modules, learning objectives, and education methods

Modules	Learning Objectives	Education Methods
Module 1	• Describe the benefits of effective clinical communication. • Understand why improving communication in Aboriginal health care is critically important. • Describe common communication barriers for Aboriginal people in health care. • Understand the relationship between Clinical Yarning, cultural security, and patient-centred communication.	Didactic presentation Small group discussion and feedback Video analysis and feedback
Module 2	• Describe skills for effective communication in Aboriginal health care.	Didactic presentation Video analysis and feedback individually and in small groups
Module 3	• Describe the Clinical Yarning framework	Didactic presentation Paired discussion and feedback Video analysis and feedback individually and in small groups
Module 4	• Identify the skills used in Clinical Yarning practice	Didactic presentation Video analysis and feedback individually and in small groups Demonstration and feedback
Module 5	• Demonstrate Clinical Yarning approach to patient communication	Simulation learning with Aboriginal community member simulation patient using Agenda Led Outcomes Based Analysis (ALOBA) method

### Evaluation

A mixed method evaluation was undertaken in order to explore the impact and processes of delivering Clinical Yarning Education to health care clinicians and students in a remote/regional area of Western Australia. The evaluation also sought to understand the perceptions of health care managers about the program and possible future implementation.

Sites, Participants and Interventions.

Five project sites were identified including the public health service (in the areas of: mental health, emergency care, remote health service), an Aboriginal Community Controlled Health Care Service, and health students on rural placements with the university department of rural health (Table 1). Participants in health services were staff who delivered clinical or support services to Aboriginal patients and/or students on placement. Staff either choose to attend the education program (emergency care, Aboriginal Community Controlled Health Care Service), or were part of a health team that had nominated to participate (mental health, remote health service). For students, it was part of their placement program (student placement).

### Evaluation – program learners

Mixed methods were used to examine the impact and processes of the Clinical Yarning Education program. Changes in learners’ perceived knowledge, confidence, competence and perceptions about communication in Aboriginal health care were ascertained using a retrospective pre-post questionnaire survey [22, 23]. Following program completion learners rated themselves before the workshop (five item scale: 1 = low, 5 = high), and then after. Retrospective pre/post evaluation, measured at one point after the program, are reported to have higher validity than before/after measures because they account for changes in participants’ perspective that might occur because of the intervention, known as response shift bias [22, 23]. The survey included demographic information (age, profession/student, previous cultural training experiences, Aboriginality) and level of agreement about program processes: how helpful learning activities were, the amount of time, how helpful for practice, and whether learners would recommend the program to others (five item scale: 1 = strongly disagree, 5 – strongly agree). The survey also included open-ended qualitative questions asking learners what was most useful about the program, and what could be improved (Appendix 1).

### Evaluation – health service managers

Qualitative semi-structured interviews were undertaken with health service managers from each site (1–2 people per organisation), who were department, section or health service team leaders from participating sites. Health service managers had not participated in the Clinical Yarning education but were directly responsible for managing staff who attended.

Interviews focussed on perceptions about the Clinical Yarning education program, current cultural training opportunities, the feasibility of future delivery of the program and support resource considerations. Interviews were supported by an interview guide (Appendix 2). Where consent was obtained interviews were recorded and transcribed. When participants did not want to be audio-recorded, written notes were taken.

### Data analysis

Quantitative data from pre post retrospective program questionnaires was entered into SPSS (IBM SPSS Statistics for Windows, Version 24.0) and descriptive statistics, including the mean, standard deviation and median for each questionnaire item, were calculated. Difference in pre post retrospective scores for all participants were determined using Wilcoxon Signed Rank Test.

Qualitative data were transcribed into Microsoft word and imported into Nvivo (version 11, QSR International) for data management. Qualitative data from survey open-ended survey questions were grouped into theme areas and enumerated. We were interested in the perspective of learners, the processes and outcomes of learning and ways in which the program may be improved from their perspective. We were particularly interested in what processes were effective for learning and why, and what could be improved. Qualitative interviews with health service managers were transcribed, read, and a thematic analysis undertaken informed by a qualitative descriptive approach. This approach aims to provide a “rich, straight description” of an experience or event [24]. When exploring the perspectives of health service managers, we considered their perspectives about Clinical Yarning, its role in staff development within their workplace, and the feasibility and considerations for further implementation of Clinical Yarning Education.

Qualitative data were reviewed by two researchers (IL and WF) who initially reviewed qualitative data and undertook a thematic analysis independently. The initial analysis framework was informed by the objectives of the interviews. Following initial independent analysis, two researchers (IL and WF) met and discussed the preliminary coding framework. Following discussion, the framework was adapted. Two rounds of analysis and coding was undertaken. Then, a written summary of findings was developed and provided to the research team. The research team acted as a ‘critical friend’ by critically reviewing the emerging findings and underlying data supporting the findings. Following this step, a final summary of findings was developed.

All methods were performed in accordance with relevant guidelines and regulations including the Declaration of Helsinki and Australian Government National Health and Medical Research Council, Ethical conduct in research with Aboriginal and Torres Strait Islander Peoples and communities [25].

## Results

### Participant evaluation

Six workshops were delivered to staff from the five sites. Thirty-eight participants undertook training, four training participants were called away urgently during workshops (e.g. were rostered on-call for health care emergencies) and did not complete an evaluation. Data are presented for 34 participants (89%) who completed the entire Clinical Yarning education program and program survey. Most participants were from mental health services, and this group included social workers, counsellors, outreach workers in community alcohol and drug services, diversion officers, and support workers (Table 3). Eight participants identified as Aboriginal and 26 participants had undertaken previous cultural training (see Table 3). Cultural training had been undertaken most commonly in the workplace (nine participants, 26%) or online (five participants, 15%). Two participants (6%) had completed cultural training overseas, in Canada and New Zealand.

**Table 3:** Participant sociodemographic characteristics

Participant characteristics	*N* = 34	
	N	%
Study site		
Mental health	12	35
Aboriginal Medical Service	4	12
Emergency Department	5	15
Rural university	6	18
Remote health service	7	21
Sex		
Female	29	85
Male	5	15
Age^a^		
18-40	15	44
41-60	16	47
61-80	2	6
Aboriginality		
Yes	8	24
No	26	76
Health profession		
Mental health	13	38
Medical	5	15
Student	6	18
Nursing	7	21
Aboriginal health practitioner/liaison officer	2	6
Dentist	1	3
Previous cultural training		
Yes	26	76
No	8	24
^b^If yes, type of cultural training		
University	4	12
Work place	9	26
Online	5	15
Overseas	2	6
Unknown/Other	3	9

^a^One missing value

^b^Partcipants could list more than one cultural training type

**Fig. 2:**
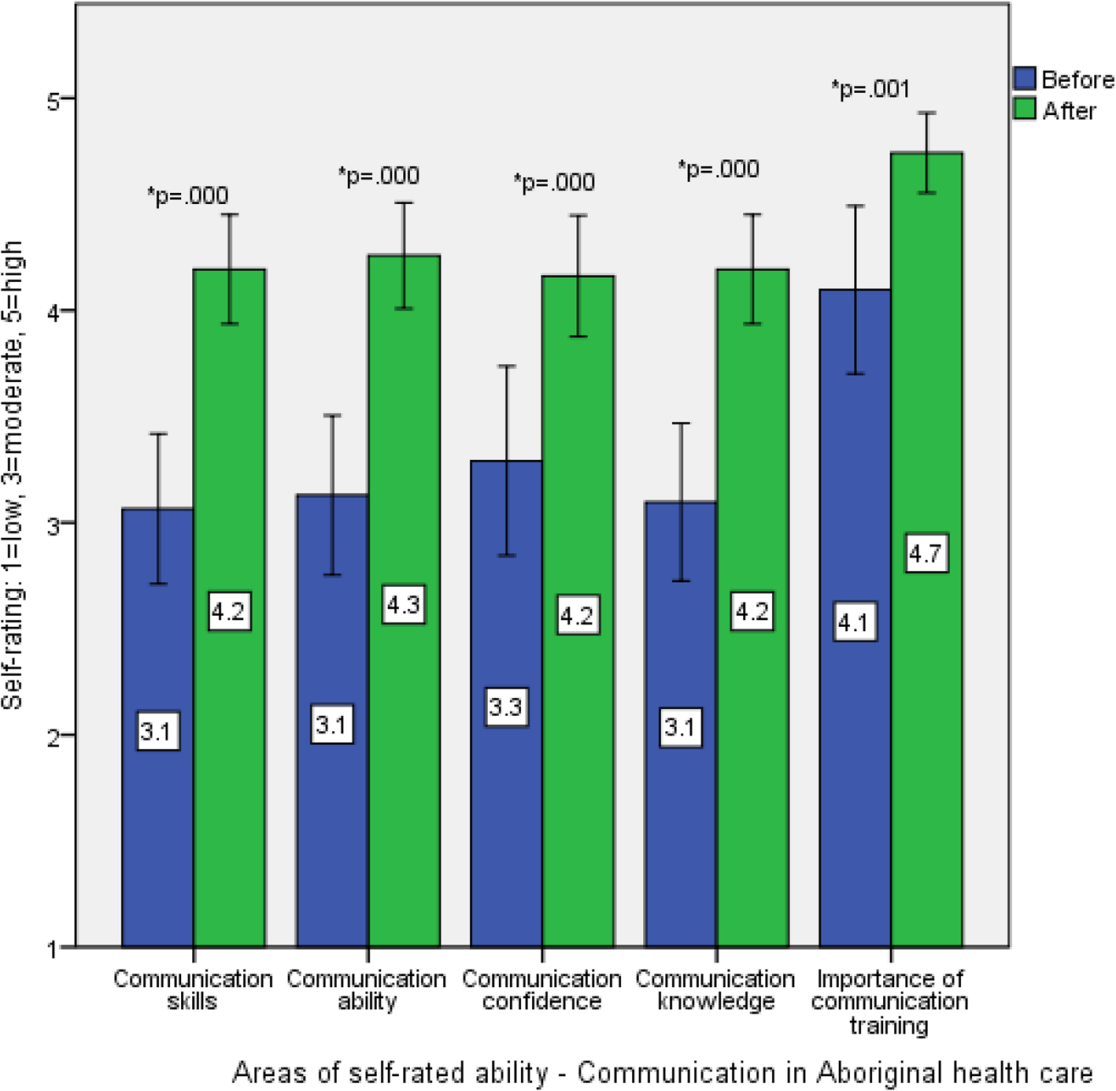
Retrospective pre/post survey results for participant self-rated skills, ability, confidence, knowledge and importance of training in Aboriginal health care communication with mean displayed (Error bars: 95% confidence interval)

There were significant improvements in retrospective self-rated communication skills in Aboriginal health care from before (M = 3.2, SD = 1.0, Md = 3) to after (M = 4.2, SD = 0.7, Md = 4) the workshop (z = 4.85, *p* = .000). Significant improvements were also noted in self-rated communication ability (before: M = 3.2, SD = 1.0, Md = 3, after: M = 4.3, SD = 0.7, Md = 4, z = 4.654, *p* = .000), confidence in communicating with Aboriginal patients (before: M = 3.3, SD = 1.2, Md = 3, after: M = 4.2, SD = 0.8, Md = 4, z = 3.841, *p* = .000), knowledge about communication in Aboriginal health care (before: M = 3.1, SD = 1.0, Md = 3, after: M = 4.2, SD = 0.7, Md = 4, z = 4.556, *p* = .000) and perceived importance of communication training (before: M = 4.1, SD = 1.1, Md = 3.5, after: M = 4.8, SD = 0.5, Md = 4, z = 3.272, *p* = .001). These are displayed in Fig. 2.

### Perceptions about the program

**Fig. 3:**
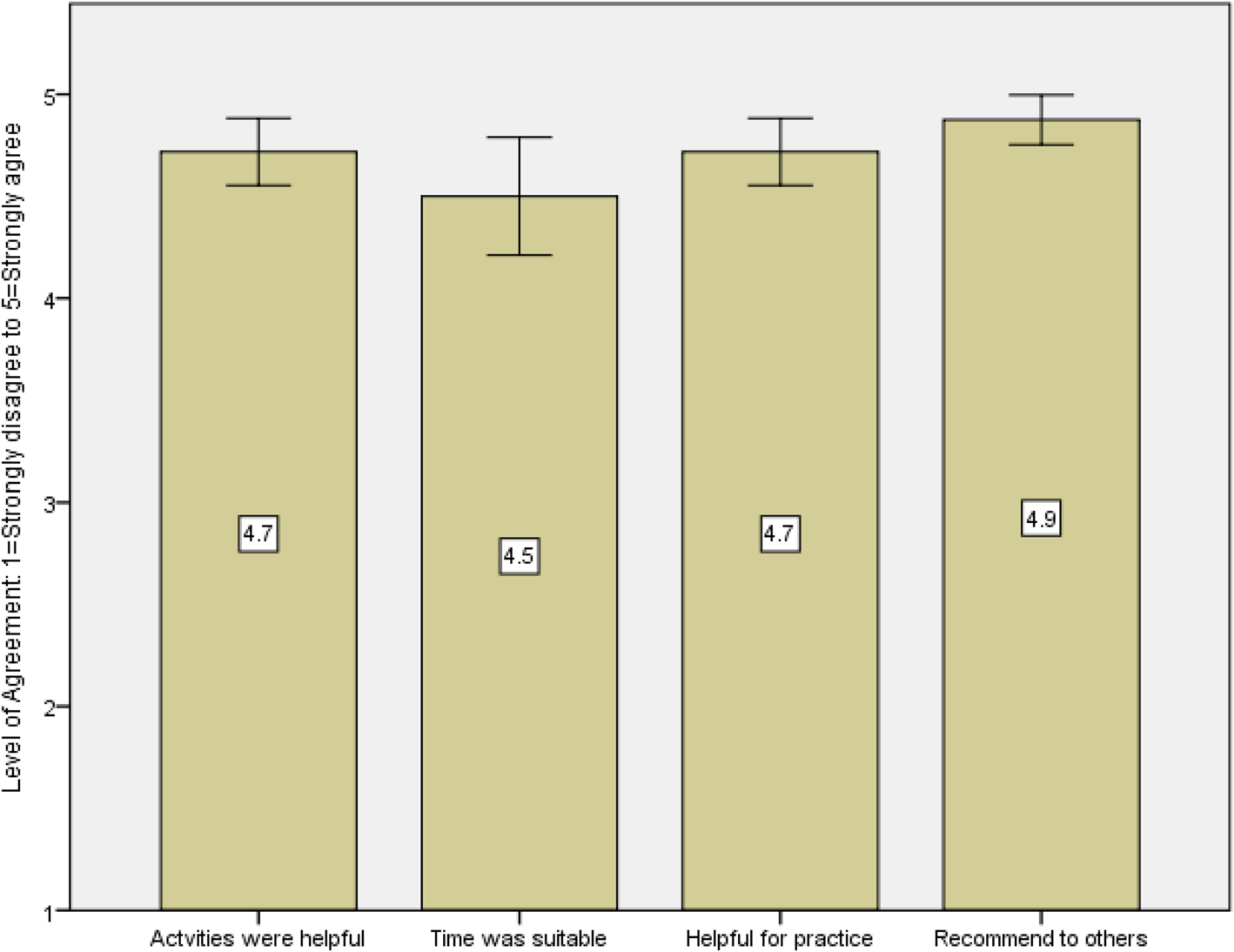
Participant agreement to program domains with mean displayed (Error bars: 95% confidence interval, scale: 1 – strongly disagree to 5 – strongly agree)

Participants had high levels of agreement regarding the helpfulness of learning activities (M = 4.7, SD = 0.5, Md = 5.0), the amount of time of the workshop (M = 4.5, SD = 0.8, Md = 5.0), the helpfulness for practice (M = 4.7, SD = 0.5, Md = 5.0), and whether they would recommend to others (M = 4.9, SD = 0.3, Md = 5.0). These are displayed in Fig. 3.

Participant perceptions about what was most useful about the program and what could be improved are illustrated with examples of qualitative data from open-ended survey questions in Table 4. The most useful aspects were the simulation/interactive aspects of the program, “all of it”, group/peer interaction and audio-visual learning resources such as videos. There were fewer suggestions for how to improve the program than suggestions for what was found to be useful. Most suggestions related to changes or additions to the simulation activity, most commonly that simulation scenarios addressed participants’ area of health care practice. Other common suggestions were ‘nothing’ and to offer follow-up or further training opportunities.

**Table 4:** Participants responses to “what did you find most useful?” and “how could the program be improved?”, the number of responses and illustrative statements

**Most useful about the program**	**Number of respondents (total no = 34)**	**Illustrative statements**
Simulation/practical learning aspects	15	*Role playing - both challenging but very useful* *Second session role play, watching then reflecting/ discussing as a group was super educational as visual learning is most effective (for me). - Able to apply theory and see do’s and don’ts.*
All/Everything	5	*All of the session* *All of it*
Peer/group interaction	4	*being critically assessed in front of peers* *The level of expertise from all different backgrounds*
Audio-visual learning resources	4	*most useful was video examples & reflection & simulated patient exercise*
Self-reflection	3	*reflect on my current strategies & be mindful of improvements I could make to my communication style, questioning*
The Clinical Yarning Framework	2	*Being provided with a framework to base my discussion/ communication with Aboriginal patients and putting it into practice.*
Expertise/skills of facilitators	2	*The knowledge provided & experienced shared*
Cultural awareness	1	*Importance of cultural awareness and importing overseas trained doctors and local doctors being exposed and educated to local customs and traditions.*
**What could be improved**	**Number of respondents**	**Illustrative statements**
Modifications/additions to simulation scenario	5	*Allowing a more realistic and specific setting - clinic/ community. Providing the scenario prior the simulated session* *More scenarios reflecting different backgrounds & issues*
Nothing	5	*Excellent* *I enjoyed it very much, found it very helpful and important*
More training/follow up session	4	*Made widespread and available to both new and old workforce. Made to be mandatory* *Maybe a follow up session to see how skills are used in “real” situations.*
Greater detail	3	*More topics to help facilitate social yarn - common talking points for Aboriginal people; not having any exposure to communicating with Aboriginal people, I felt unsure what to talk about.* *Breakdown of each area to more steps – explain Social Yarning ice breakers*
More videos/examples	2	*Perhaps real life stories from Indigenous people of negative experiences regarding - social, diagnostic and management yarning for stronger understanding. (There is effect in hearing stories from people you know/ work with/ in front of you compared to a clip acted out). Learnt more than expected! Thanks! :)*
More time	2	*More time to allow for further discussion*
More practice	1	*More practice sessions*

### Interviews – health service managers

Six health care managers, at least one from each health site, were interviewed. Managers had professional backgrounds in medicine, nursing (three participants), social work, and Aboriginal health. Five interviews were recorded and transcribed verbatim. One health service manager preferred not to be audio recorded and written notes were taken. Interviews lasted between 20 and 40 min. Interviews were centred around three major themes; (i) existing cultural training, (ii) about Clinical Yarning, and (iii) future implementation considerations. Key findings related to each theme and subtheme are presented below with examples of illustrative statements in Table 5.

**Table 5:** Themes and subthemes from interviews with health care managers, including illustrative statements as examples

**1. Existing cultural training**
*Other than that* (online cultural training), *to be honest not really much else happens* (Participant 5) *…that’s* (online cultural training program) *quite broad, it’s not particularly clinical… that’s just… generic for everybody* (Participant 5) *We have cultural awareness training, which happens every month. That’s delivered by XXXX.* (Participant 2)
**2. About Clinical Yarning**
The Clinical Yarning Concept 2a. *There is a big difference in the communication style between clinicians and Aboriginal patients and the information that Aboriginal people need* (Participant 1) *It was something that was never heard of before to be honest. You know it doesn’t come through medical school, it doesn’t come through you know your junior doctor training or anything. So it’s completely new… So it’s something that we just don’t get any sort of exposure to. So yeah, no, I thought it was good, because I’ve never seen it before*. (Participant 6)
Clinical Yarning training 2b. *…there’s been positive feedback. So, there’s nothing here that’s come back negative at all. So, they do feel that it was worthwhile and that they’re glad that they did it, yes*. (Participant 2) 2c. *So, I think the practical component is a big emphasis on making them feel secure in the workplace and actually giving them those skills to actually defuse situations that they’re faced with, and how to communicate more effectively with the client. Because sometimes they’re taught about asking questions that are in a care plan, but there’s a way of asking those questions to people to get a better response*. (Participant 4) *The staff who have done it really have found it very helpful, and practical actually for what we do with our clients*. (Participant 5)
**3. Future Implementation Considerations**
Training options 3a. *ELearning gives options to our staff who are out in outer regions who can’t get in to town to do it. I think in this day and age we do need to have a few different options for training*. (Participant 5) 3b. *One thing - good thing that computer training is very good at, is repeating the same information over and over again, and people can do it at their leisure, and then being able to do the practical stuff face to face*. (Participant 3) 3c. *Yeah, look I’m in two mixed minds about that sort of half online half practical stuff. I mean a lot of our courses seem to be going down that way of doing, you know, some of I suppose the bookwork online and then reducing the face-to-face time. I think some people respond to it. Some people don’t respond to the online stuff. They don’t do it. They put it off and when they do they just click next, next, next, next, next and don’t bother actually reading it*. (Participant 6)
Train-the-trainer model 3d. *The partnership model of facilitation* (with an Aboriginal and clinical facilitator) *works well.* (it) *benefits Aboriginal staff because it is not just them saying there is an issue…* (it) *increases the confidence of Aboriginal staff to talk up about issues* (Participant 1) *The more we can do at home base the more I’d support it. So, to have a local person trained effectively to deliver this training to me would be beneficial, because the challenge we have is that we depend a lot with other training on people coming out… So, the more self-sufficient we can be at the workplace the better it works for us really* (Participant 4) 3e. *I know you met [staff member] who’s our Aboriginal liaison officer. I know she’s very keen on cultural awareness training and any aspects of that…. So for somebody, for example, like her that is an Aboriginal person that’s talking about Aboriginal issues and part of her role is facilitating and educating Aboriginal people to come to their medical appointments and supporting them to do so - somebody like her would be a key component to have as a trainer to provide ongoing education to our new employees as part of their orientation to site*. (Participant 4) *I mean we might be lucky, if you put it out there then you might find someone who is really keen and wants to take it on, but I wouldn’t count on that*. (Participant 6)
Future Implementation Considerations Opportunities 3 f. *We could introduce it at orientation level. Locums who come visit us all the time, you may have a shortened version that we could introduce to the locums at orientation. Bearing in mind that they are maybe here for a week or two weeks but we do need to have the first half a day for locums to be training. That could be slotted into something that is mandatory orientation*. (Participant 2) *Implementing CY would be helped if mandated through regional executive or WACHS board… maybe as a KPI… this would add weight and reduce barriers* (Participant 1) 3 g. *I think good engagement with the Aboriginal workers on the ground is really useful, because they’re the ones that will be promoting it in the service, and they’ll know, when you’re having that conversation, they’re able to draw on that when they’re having the corridor conversations, or the clinical reviews, as well, in that process. Some top-down support doesn’t hurt either, as well*. (Participant 3) [Senior manager] *would be a good ally locally* (Participant 1) Challenges 3 h. *I think one of the main hurdles for us will be our transiency of staff because that’s something that we have to take into consideration; that whoever might be chosen to do it now we’ve always got to have good succession planning in place for those people should they choose to move on*. (Participant 4) *I mean I think the problem you’re going to have once you go to the smaller sites is a problem we have here, is just getting the staff off the floor to attend. We’re lucky we’ve got slightly more staff so we have more staff off-duty who can come in. You go to [remote site] or somewhere like that where there’s only four or five docs, you’re basically going to have to deliver it almost one-on-one or one-on-two, because the other two will be working. Then try and get them when they’re not on days off and going away somewhere and all that. Logistically it just becomes challenging.* (Participant 6)

#### Existing cultural training

Health service managers from the state government health service indicated that, with the exception of a generic, mandatory online cultural training package for all state government health care employees, face-to-face cultural training was ad hoc in nature and delivered by Aboriginal staff when a need arose, generally only if there were Aboriginal staff within the department (Table 5:1). Most participants indicated that online cultural training did not meet the needs of clinicians because of its general nature, with some participants questioning the relevance to clinical practice.

This contrasted to the Aboriginal Community Controlled Health Care Service which provided regular face-to-face cultural training (Table 5:1, participant 2).

#### About clinical yarning

Participants discussed two subthemes, relating to the Clinical Yarning concept, and the Clinical Yarning training.

Participants were universally supportive of the Clinical Yarning concept. A common theme was that Clinical Yarning addressed a current gap; including both a gap in communication between Aboriginal patients and clinicians and a gap in existing cultural education. The uniqueness of the Clinical Yarning approach was highlighted as filling this gap (Table 5:2a).

A second substantive subtheme related to the Clinical Yarning training provided. All participants reported strong positive feedback about Clinical Yarning training from staff within their areas (Table 5:2b). About half of participants highlighted positive feedback received from staff, including the practical focus of training, and that a skills-based approach to training was considered useful for clinical practice (Table 5:2c).

#### Future implementation considerations

All participants strongly supported continued delivery of Clinical Yarning education. Opportunities for future implementation included offering alternate training options, a train-the-trainer model, and partnerships. Challenges related mainly to staff shortages and transiency, especially in remote regions.

Some, but not all participants, indicated that having alternate training options would be helpful, especially the option for part of the training to be offered via eLearning. The flexibility of eLearning was seen as useful to some learners, especially those in remote areas (Table 5:3a). Others highlighted that eLearning was only useful if accompanied by face-to-face, practice-based training as well (Table 5:3b), whilst one participant exclusively supported face-to-face training instead of a combination of eLearning and face to face training (Table 5:3c).

A train-the-trainer model, in which staff from within health care services were trained and supported to deliver Clinical Yarning education programs ‘in-house’, was highlighted by most participants. Some of the benefits discussed included building the capacity of Aboriginal and non-Aboriginal staff to deliver in partnership, and being able to meet training needs ‘self-sufficiently’ (Table 5:3d). This could be enhanced by ensuring adequate support for trainers and linking training of Aboriginal staff as Clinical Yarning facilitators to a recognised qualification, such as a Certificate IV in Training and Assessment offered through Technical and Further Education institutions.

Half of health managers interviewed discussed the importance of identifying the right person for a trainer role. Some participants readily identified specific people from within their workplaces who they felt were suitable, whilst others highlighted how identifying the right person could be a challenge (Table 5:3e).

Participants identified a number of strategies to support future Clinical Yarning education programs. Suggestions included Clinical Yarning as a key performance indicator, including as part of orientation for new staff, or including within existing training systems/approaches such as professional development programs (Table 5:3f). Although some suggested mandating Clinical Yarning training for all staff, making a program “mandatory” was viewed by one staff member as potentially off-putting.

A significant subtheme relating to future implementation was building partnerships and allies within health care services. Participants recommended developing partnerships at multiple levels; with managerial staff, Aboriginal staff ‘on-the-ground’, and identified leaders from within health settings (Table 5:3g).

The primary challenge discussed related to staff resourcing, especially in remote areas. These included transiency/turnover of staff (if there were a train-the-trainer approach), and the difficulty of scheduling training when staff are required to work in low-staffed environments (Table 5:3h).

## Discussion

Clinical Yarning is a patient-centred communication framework for Aboriginal health care. This study adds to recent evidence [37], from the perspective of HCPs and health students, for a Clinical Yarning education program. Whilst there is more research to do, results from this study provide a foundation for further implementation work and investigation of the impact of Clinical Yarning on health care practice, the health care experiences of Aboriginal people, and health outcomes.

The Clinical Yarning educational program developed in this project was underpinned by several important principles. Firstly, cultural security was an over-arching principle that ensured the project did not compromise the ‘legitimate cultural rights, views, values and expectations of Aboriginal people’ [26]. In practice this was seen as close partnerships between Aboriginal and non-Aboriginal people at each stage and level of the work within and beyond the research team, resulting in co-design of project materials. The research group included expertise in Aboriginal culture, Aboriginal health research, clinical practice, and patient-centred communication and education. Educational workshops were co-facilitated by staff with Aboriginal cultural knowledge and health care clinical practice. Given that Clinical Yarning is an approach used at the interface between Aboriginal people and their interaction with the health care system, principles of cultural security, partnerships, co-design and co-facilitation are essential for future Clinical Yarning implementation.

The training workshop applied evidence-informed education principles for delivering patient-centred communication skills training. These principles, including behavioural skills-based learning strategies that were interactive, involved role-play, feedback, and small group discussion [20, 21] and based on an adult learning approach [19], were highly valued by participants. Simulation learning with Aboriginal community members who were trained as simulation patients was also valued by participants and recommended when training clinicians in patient-centred communication skills [10]. Participants suggested that simulation learning could be enhanced by including simulation scenarios specific to the work of clinicians being trained. Clinical yarning training tailored to a specific area of health care has recently been reported in the area of persistent pain [27].

Communication is at the heart of culturally safe health care [28] and most interventions to improve cultural security/safety in health care involve education. Contemporary cultural practice frameworks are multidimensional and include practitioner attitudes and values, skills, knowledge/experiences, and actions [29]. To our knowledge, Clinical Yarning is the first culturally oriented education program that applies a skills-focus to cultural practice. Clinical Yarning education is not cultural awareness/safety/competence training although the focus on skills could compliment cultural awareness training that addresses knowledge, attitudes and values. This approach is embedded within student programs at the Western Australian Centre for Rural Health, Australia, in which students on rural placement attend local cultural training, ‘Miyarnuwimanha’ and then Clinical Yarning training [30]. This approach has also been recently reported amongst pain clinicians in Queensland, Australia, in which a training program that included first cultural capability, and then Clinical Yarning was implemented [27].

Implementing new approaches in health care can be challenging. In our study further implementation of Clinical Yarning was supported by all health service managers who were interviewed. To facilitate implementation, flexible training options such as eLearning for part of the training, training Clinical Yarning facilitators from within health care services in a train-the-trainer model, and incorporating Clinical Yarning into existing orientation or professional development programs was recommended. Another enabler highlighted was to adopt a multilevel approach by engaging and building alliances at both clinician and managerial levels within health services. These factors will form the basis of future interventions. Additional considerations include systemic and organisational factors such as supportive policy, committed leadership from within health services, user engagement (i.e. involvement of Aboriginal consumers), organisational readiness, delivery across multiple sites, and use of audit and quality improvement approaches [31, 32].

A limitation of these findings is that our evaluation only focussed on the perspective of health practitioners and ultimately, understanding the effect on health care practice, the patient experience and health outcomes is ideal. Whilst like ours, some clinician training interventions to improve patient-centred communication are a half a day or less, they also included other interventions to change practice behaviours such as clinical tools and reminders [33, 34]. Other communication training interventions are longer, such as 3–4 days in total [35, 36]. Further investigation to understand what ‘dose’ of Clinical Yarning education is needed to influence practice and what supportive tools may assist is needed. Future implementation research should include an investigation of health system, health service and clinician barriers and enablers to uptake of the Clinical Yarning approach and Clinical Yarning education program so interventions can be developed addressing these. The program was delivered in one region and how it translates to elsewhere is unknown, although the experience of investigators and recent research suggests that Clinical Yarning translates to other areas in Australia [37]. Finally, we do not know to what degree participants represent those who are self-selected and more open to training of this nature. However, results were consistent across the different health/university settings, included participants who had, and who had not received previous cultural training, and there was strong alignment between program participants and health care manager perspectives. This supports the generalisability of outcomes, although further investigation is needed.

## Conclusion

A four-hour, face-to-face, skills-based Clinical Yarning education program delivered to clinicians and students from varied health care professions resulted in increased self-reported skills, ability, confidence, and knowledge about communicating in Aboriginal health care and was well received by participants. The program was also valued by health care managers. Future implementation into health care could consider flexible learning options for part of the program (such as eLearning) and a train-the-trainer approach to train health service staff as Clinical Yarning facilitators. This study provides preliminary support for the Clinical Yarning education program and provides the foundation for further examination of this model.

### Supplementary Information

**Additional file 1: Appendix 1.** Retrospective pre/post survey. **Appendix 2.** Interview/Yarning Guide Departmental and Organisational Representatives

## Data Availability

The datasets used and/or analysed during the current study are available from the corresponding author on reasonable request.
