# Supplementary material for: Clinical yarning education: development and pilot evaluation of an education program to improve clinical communication in Aboriginal health care - participant, and health manager perspectives
Source: BMC Med Educ. 2023 Nov 30;23:908. doi: 10.1186/s12909-023-04843-8 (PMC10688002; doi:10.1186/s12909-023-04843-8)
Supplement: Supplementary file 1 — Additional file 1: Appendix 1. Retrospective pre/post survey. Appendix 2. Interview/Yarning Guide Departmental and Organisational Representatives [file 12909_2023_4843_MOESM1_ESM.pdf]

Appendix 1: Retrospective pre/post survey

Clinical Yarning Education Program

1. Are you completing this program as a student? Yes ☐ No ☐

2. What is your profession/area of study/area of work? ____________________

3. What is your age (in years)? ________________

4. What is your sex? Male ☐ Female ☐ Other ☐

5. Do you identify as Aboriginal or Torres Strait Islander? Aboriginal ☐ Torres Strait Islander ☐ Both ☐ Neither ☐

6. Where are you undertaking this training? XXXXXX ☐ XXXXXX ☐ XXXXXX ☐ XXXXXX ☐ XXXXXX ☐ Other, please list: ______________

7. Have you had previous cultural training? No ☐ Yes ☐ if yes, please describe: _____________
____
____
____

8. Please circle one rating for both *before* and *after* this workshop:

	Low		Moderate		High
a. How would you rate your level of skill to communicate with Aboriginal patients:					
Before this workshop	1	2	3	4	5
After this workshop	1	2	3	4	5
b. How would you rate your ability to communicate with Aboriginal patients					
Before this workshop	1	2	3	4	5
After this workshop	1	2	3	4	5

Western Australian Centre for Rural Health

Government of Western Australia WA Country Health Service

THE UNIVERSITY OF WESTERN AUSTRALIA

WAPHA WA Primary Health Alliance

c. How would you rate your confidence to communicate with Aboriginal patients:					
Before this workshop	1	2	3	4	5
After this workshop	1	2	3	4	5
d. How would you rate your knowledge of patient-practitioner communication in Aboriginal health care:					
Before this workshop	1	2	3	4	5
After this workshop	1	2	3	4	5
e. How would you rate the importance of communication training for clinicians in Aboriginal health care:					
Before this workshop	1	2	3	4	5
After this workshop	1	2	3	4	5

9. Please rate your agreement with the following:

	Strongly Disagree	Disagree	Undecided	Agree	Strongly Agree
a. The learning activities were helpful					
b. The amount of time was suitable					
c. The program was helpful for practice					
d. I would recommend this program to others					

10. What did you find most useful?

11. How could the program be improved?

Appendix 2: Interview/Yarning Guide Departmental and Organisational Representatives

Yarning Guide – Departmental and Organisational Representatives

Purpose

The purpose of this research yarn is to explore organisational perspectives about training local health staff to facilitate Module 5 of the Clinical Yarning Education program and the feasibility of implementing this training into the future.

Guide

Can you describe how Clinical Yarning Education fits with education/training activities of your department? (Prompts: what other education/training is there? how often does training/support occur? Is Clinical Yarning education valued? Why/why not?)

Describe model of Clinical Yarning education – eLearning, training health staff as facilitators of module 5. How effective do you feel this approach has been? (Prompts: Why/why not? Examples/stories to illustrate)

How feasible is implementing Clinical Yarning Education in your department/other regions in the future? (Prompts: What could help this? What could hinder? How could this be better?)

What else?
